# Shifting from vitamin K antagonists to non-vitamin K antagonist oral anticoagulants in patients with atrial fibrillation: predictors, patterns and temporal trends

**DOI:** 10.1186/s12872-021-02295-w

**Published:** 2021-10-13

**Authors:** Arthur Shiyovich, Varda Shalev, Gabriel Chodick, Matanya Tirosh, Amos Katz, Miriam M. Klar, Mony Shuvy, David Pereg, Sa’ar Minha

**Affiliations:** 1 Department of Cardiology, Rabin Medical Center, 39 Jabotinsky St., 49100 Petah Tikva, Israel; 2 Sackler School of Medicine, Tel Aviv University, Tel Aviv, Israel; 3 Maccabi Healthcare Services, Kahn-Sagol-Maccabi Research and Innovation Institute, Tel Aviv, Israel; 4 Medical affairs, Pfizer, Israel; 5 Faculty of Health Sciences, Ben-Gurion University of the Negev, Beer-Sheva, Israel; 6 Department of Medicine, SUNY Downstate Health Sciences University, Brooklyn, NY USA; 7 Department of Cardiology, Shamir Medical Center, Zerifin, Israel; 8 Heart Institute, Hadassah Hebrew University Medical Center, Jerusalem, Israel; 9 Department of Cardiology, Meir Medical Center, Kfar-Saba, Israel

**Keywords:** Atrial fibrillation, Non-vitamin K antagonist oral anticoagulants, vitamin-k antagonists, Time to initiation, Shifting

## Abstract

**Background:**

Non-Vitamin K antagonist oral anticoagulants (NOACs) emerged as an alternative with comparable or superior efficacy and safety to vitamin K antagonists (VKAs) for stroke prevention in patients with non-valvular atrial fibrillation (AF).

**Objectives:**

The aim of the current study was to investigate the patterns, predictors, timelines and temporal trends of shifting from VKAs to NOACs.

**Methods:**

In this retrospective observational study, the computerized database of a large healthcare provider in Israel, Maccabi Healthcare Services, was searched to identify patients with AF for whom either a VKA or NOAC was prescribed between 2012 and 2015. Time from diagnosis to therapy initiation and to shifting between therapies was evaluated.

**Results:**

Out of 6987 eligible AF incident patients, 2338 (33.4%) initiated treatment with a VKA and 2221 (31.7%) with a NOAC. In addition, 5259 prevalent patients were analyzed. During the study period, NOAC prescriptions proportion among the newly diagnosed cases increased from 32 to 68.4% (*p* for trend <  0.001). The median time from diagnosis to first dispensing was greater in NOAC than VKA and decreased among patients treated with NOAC during the study period (2012: 1.9 and 0.3 months, 2015: 0.7 and 0.2 months, respectively). During follow-up, 3737 (49%) patients (54.3% and 47.1% of the incident and prevalent cases, respectively), shifted from a VKA to a NOAC, after a median of 22 months and 39 months in the incident and prevalent cases, respectively, decreasing throughout the study period. Female gender, younger age, southern district, higher CHADS_2_ and CHA_2_DS_2_-VASC score, non-smoking, and treatment with antiplatelets were associated with a greater likelihood for therapy shift. Shifting from a NOAC to a VKA decreased over time from 8 to 4.5% in 2012 to 0.5% and 0.7% in 2015 in the incident and prevalent groups, *p* <  0.001 respectively.

**Conclusions:**

Shifting from VKA to NOAC occurred in 50% of the cases, more frequently among incident cases, and younger patients with greater stroke risk. Shifting from a NOAC to a VKA was much less frequent, yet it occurred more often in incident cases and decreased over time. A socially and economically sensitive program to optimize the initiation of OAC therapy upon diagnosis is warranted.

**Supplementary Information:**

The online version contains supplementary material available at 10.1186/s12872-021-02295-w.

## Introduction

Atrial fibrillation (AF) is the most frequent cardiac arrhythmia, with greater prevalence in older persons and patients with comorbidity such as diabetes mellitus, hypertension, heart failure, chronic kidney disease, and valvular heart disease [1, 2]. AF is associated with an approximately 5-fold increase in the risk of stroke, accounting for every fifth ischemic stroke [1, 3, 4], which may result in a poor quality of life, morbidity, and mortality [5–8]. Furthermore, delayed initiation of therapy associated with substantial stroke risk was reported [9]. For many years, vitamin K antagonists (VKA) were the cornerstone for stroke prevention in patients with AF. However, their use is limited by the need for frequent monitoring, a narrow therapeutic range, and a restricted diet, which often results in reduced compliance and persistence with therapy [10–12]. Non-Vitamin K antagonist oral anticoagulants (NOACs) emerged as an alternative with comparable or superior efficacy and safety to VKAs for stroke prevention in patients with AF. Furthermore, NOACs have fixed dosing regimens, do not require routine monitoring or significant diet restrictions [13–15]. Thus, treatment guidelines prefer NOACs over VKAs in patients with AF; however, routine shifting of stable VKA-experienced patients is not recommended [16–18]. Although NOACs were rapidly adopted as initial therapy based on reports derived from registries and guidelines, they often remain underutilized and often delayed or among patients with AF [9, 16, 19–25]. Previous studies have shown a 10–70% shifting rate from VKA to a NOAC [19, 26, 27] associated with younger age, female gender, eGFR ≥ 60 ml/min, higher CHA_2_DS_2_-VASC score, and specific comorbidities (stroke, bleeding, heart failure, and alcohol abuse). However, data dealing with initiation and shifting patterns and timelines remain scarce. The current study aimed to investigate patterns, predictors, timelines, and temporal trends of shifting from VKAs to NOACs.

## Materials and methods

### Study design and population

The present retrospective observational analysis utilized real-world data of the Maccabi Healthcare Services (MHS), one of four major state-mandated healthcare providers in Israel, serving 2.3 million members.

The database search for inclusion criteria included all patients insured by MHS at the time of the dataset closure. Patients considered for inclusion in the study were those 21 years of age or older with a documented diagnosis of AF (International Classification of Diseases version 9 [ICD-9] code 427.3x) who met at least one of the following criteria: (a) AF documented in either a hospital discharge form or cardiologist visit (b) two documented AF -related visits by a primary care physician, or (c) AF documented by any other physician and a documented prescription for either a VKA or NOAC. Eligible patients not prescribed either VKA or NOAC were not included. Of these patients, those with new diagnoses of AF during the screening timeframe of January 1, 2012, to December 31, 2015, who had never received oral anticoagulation therapy were included in the study and labeled the “incident cases” group. Patients, who had received the diagnosis of AF and had already been treated with VKAs for at least six months before January 1, 2012, were also included in the study and labeled as the “prevalent cases” group. The date of AF diagnosis was defined as the index date. Exclusion criteria: patients with known congenital heart disease, valvular heart disease, active malignancy, pulmonary embolism within six months from the index date, or pregnancy ending within 16 months before AF diagnosis. Follow-up continued until July 2016.

The study protocol was approved by the Maccabi Healthcare Services Institutional Review board (0046-16-BBL) and conducted in accordance with the Helsinki declaration. Patient consent was waived by the review board.

Israel has a National Health Insurance Law which mandates medical insurance for all citizens. Penetration of NOAC to the market was thus dependent on government approval. Medications covered under the “health services basket” are determined yearly by Israel’s Ministry of Health, and copayment for these medications is minimal. NOACs were included in this basket since 2011, but patients prescribed NOACs who did not meet the basket’s criteria could purchase these drugs with higher copayment based on their insurance plan. The criteria and indications stated in the yearly “health services basket” for NOACs were broadened over the years. NOACs can be prescribed by any physician, but the copayment necessitates an approval of the healthcare provider.

### Data collection and definitions

MHS databases were used to collect patient and physician demographics. Socioeconomic status (SES) was derived from these databases by utilizing a commercial index (Points Location Intelligence, Ramat Gan, Israel) which was previously shown to highly correlate with the SES index provided by Israel’s National Bureau of Statistics [28]. Baseline comorbidities and risk score calculations were based on automated patient registries maintained by MHS, such as the diabetes mellitus [29] and cardiovascular diseases registries [30]. These registries are updated daily and automatically utilizing strict algorithms. Prescribing physician data, including birth year, sex, country of medical education attainment, and specialty, were collected from MHS’s human resources database.

The shift in therapy was defined based on dispenses. In addition, the patient had to have > 90 days of follow-up after the first dispense in that year to be eligible for this analysis. The time between AF diagnosis and first dispense (initiation) and the time from dispense of one group to dispense of a different group (shift) were collected and analyzed.

### Statistical analysis

Patient characteristics were described by means, standard deviations (SD), and frequency (%). Intergroup comparison utilized the Mann-Whitney and two-sample t-test to compare between non-normally and normally distributed continuous variables. The Chi-square test was used for categorical variables comparison. The Kolmogorov–Smirnov method was employed to test for normal distribution. The Extended Mantel–Haenszel test for linear trend was used to assess trends in NOAC prescription between 2012 and 2015. Time to treatment shift from VKAs to NOACs, both in the prevalent and the incident populations, was described with Kaplan–Meier survival plots. Multivariate logistic regression was utilized to explore independent predictors of shifting. All variables introduced in the model were chosen based on clinical relevancy (forced model). Statistical analyses were performed with IBM SPSS Statistics for Windows, Version 25 (IBM corp. Armonk, NY). The study sample size allowed for 80% power for detecting variables associated with a shift to NOACs with a minimal adjusted odds ratio of 1.15 and a significance level of 0.05.

## Results

### Study cohort and initial prescription patterns

A total of 6987 eligible incident patients were identified throughout the study period. Of them, 2338 (33.4%) initiated treatment with a VKA and 2221 (31.7%) with a NOAC. In addition, 5259 prevalent patients were eligible for the current study. Baseline characteristics of the included patients and comparisons between patients first initiated with VKA vs. NOAC are presented in Table 1. Patients who were initially prescribed NOACs were older, more likely to be females, had higher socioeconomic status and CHA_2_DS_2_-VASc score and were less likely to be immigrants. Additional file 1: Figure S1 displays the temporal trends in the relative rates of the initially prescribed medication (NOAC vs. VKA) among the incident cases between 2012 and 2015. A statistically significant increase in the NOAC prescription rate was observed, from 32% to 2012 to 68.4% in 2015, with a corresponding decrease in the VKAs rate (*p* for trend <  0.001).

**Table 1:** Baseline characteristics of the study cohort; comparison between incident cases first prescribed VKAs to those first prescribed NOACs

	Incident cases 2012–2015: First treatment prescribed after diagnosis (N = 4559)		*p*-value	Prevalent patients*
	VKA N = 2338 % (n)	NOACs N = 2221 % (n)		VKA N = 5259 % (n)
Years from NVAF diagnosis to 1/1/2012 Median (IQR)				7.59 (5; 9.5)
Age (years), Mean (SD)	70.8 (10.7)	72.8 (11.4)	**< 0.001**	74.5 (9.7)
Median (IQR)	71.6 (64.2; 78.7)	74.4 (65.9; 80.8)		75.2 (68.2; 81.6)
Female sex	43.3% (1012)	46.5% (1033)	**0.031**	45.7% (2402)
Immigration ≥ 1990	40.5% (946)	28.0% (622)	**< 0.001**	38.7% (2036)
SES score, Mean (SD)	5.74 (1.75)	6.44 (1.85)	**< 0.001**	5.93 (1.78)
Median (IQR)	6 (5; 7)	6 (5; 8)		6 (5; 7)
District				
North	18.3% (429)	18.5% (410)	**< 0.001**	18.3% (960)
Sharon	17.3% (405)	18.7% (416)		17.6% (924)
South	20.6% (481)	14.4% (320)		19.9% (1049)
Center	19.2% (448)	27.2% (604)		20.0% (1051)
Jerusalem/ Hashfela	24.6% (575)	21.2% (471)		24.2% (1275)
CHADS_2_ score Mean (SD), Median (IQR)	1.80 (1.17) 2 (1; 2)	2.03 (1.32) 2 (1; 3)	**< 0.001**	2.42 (1.30) 2 (2;3)
CHADS_2_ risk levels				
0	11.8% (276)	12.0% (266)	**< 0.001**	4.4% (234)
1	30.3% (709)	24.8% (550)		19.9% (1046)
≥ 2	57.9% (1353)	63.3% (1405)		75.7% (3979)
CHA_2_DS_2_ VASC score Mean (SD)	3.27 (1.70)	3.67 (1.88)	**< 0.001**	4.01 (1.66)
Median (IQR)	3 (2; 4)	4 (2; 5)		4 (3; 5)
Baseline comorbidity				
Congestive heart failure	9.0% (211)	9.0% (199)	0.98	25.9% (1363)
Ischemic heart disease	24.9% (582)	28.6% (636)	**0.005**	39.2% (2060)
Myocardial infarction	12.4% (289)	12.1% (268)	0.796	15.0% (790)
Cerebrovascular accident	8.0% (188)	12.1% (268)	**< 0.001**	15.9% (838)
Transient ischemic attack	4.3% (100)	5.9% (132)	**0.013**	8.2% (431)
Peripheral arterial disease	5.6% (131)	5.2% (115)	0.569	9.0% (472)
Chronic kidney disease	33.4% (782)	37.1% (824)	**0.011**	50.0% (2628)
Diabetes mellitus	33.0% (771)	36.6% (813)	**0.011**	38.5% (2025))
Hyperlipidemia	80.0% (1871)	84.7% (1882)	**< 0.001**	88.5% (4654)
Hypertension	78.4% (1834)	78.7% (1747)	0.888	86.1% (4530)
Chronic obstructive pulmonary disease	2.7% ( 62)	2.9% ( 65)	0.636	3.3% (172)
Concomitant medications**				
ACE inhibitors	26.8% (627)	26.7% (594)	0.982	37.7% (1983)
ARBs	24.3% (568)	28.5% (634)	**0.001**	61.5% (3232)
Beta blockers	38.5% (901)	38.7% (859)	0.947	10.1% (533)
Ca channel blockers	33.2% (777)	33.9% (753)	0.654	49.6% (2611)
Diuretics	29.2% (682)	29.3% (651)	0.943	5.2% (274)
Nitrates	2.8% ( 65)	3.0% ( 67)	0.698	0.1% ( 4)
Aspirin	40.7% (951)	42.0% (932)	0.394	21.3% (1122)
Other Antiplatelets	6.8% (160)	12.1% (268)	**< 0.001**	1.6% ( 84)

*At least 2 dispenses in 120 days prior 1-JAN-2012. The status of all time-dependent parameters is shown for 01/01/2012

*VKA* vitamin K antagonists, *NOAC* new oral anticoagulants, *SES* socioeconomic status, *ACE* angiotensin converting enzyme, *ARB* angiotensin receptor blocker, *SD* standard deviation, *CHADS* congestive heart failure, hypertension, age > 75, diabetes mellitus, stroke/transient ischemic event; scale of 0 (lowest risk) to 6 (highest risk). CHA2DS2-VASc: Congestive heart failure, hypertension, age, diabetes mellitus, stroke/transient ischemic event, sex, vascular disease history; scale of 0 (lowest risk) to 9 (highest risk)

Bold means statistically significant, i.e. *p* < 0.05

As presented in Fig. 2, the median time from diagnosis to first dispensing was greater in NOACs than VKAs; however, it decreased among patients treated with NOACs throughout the investigated period.

### Shifting therapy

**Fig. 1:**
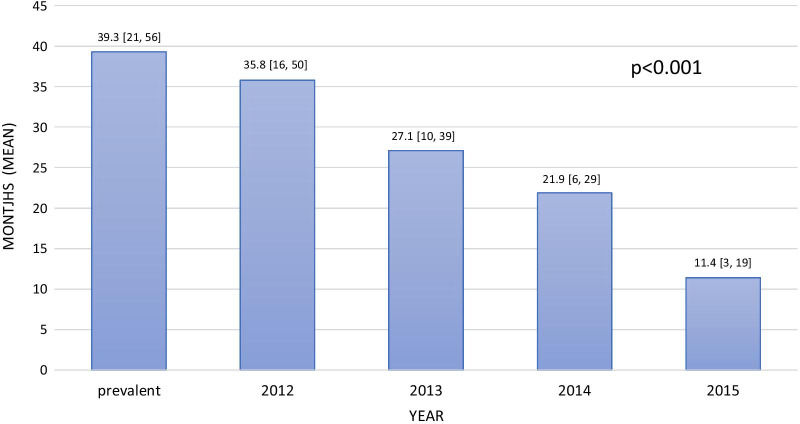
Time from initiation of treatment to shift (months, mean [IQR]) from VKA to NOACs

**Fig. 2:**
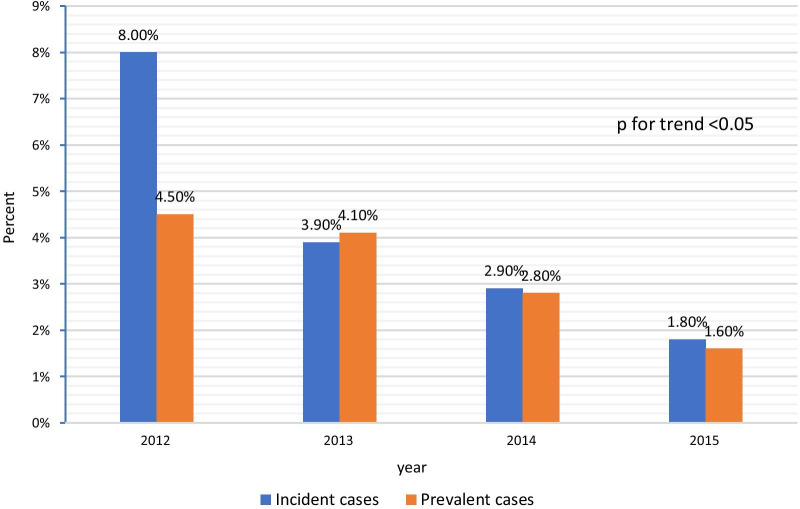
Time from initiation of treatment to shift (months, mean [IQR]) from VKA to NOACs

During follow-up, 3737 (49%) patients (54.3% and 47.1% of the incident and prevalent cases, respectively), initially prescribed with VKAs, shifted to NOACs. The clinical characteristics of patients, who shifted from VKAs to NOACs compared with those who did not, are presented in Table 2. Patients who shifted were older, more likely to be females, had a greater prevalence of congestive heart failure, diabetes mellitus, hypertension, hyperlipidemia, higher CHADS_2_ and CHA_2_DS_2_-VASC score, and were more frequently incident cases versus those that did not shift. The median time from initiation of therapy to shifting was 22.1 months [IQR, 8.3–37.6] and 39.3 months [IQR, 20.7–55.8] for the incident and prevalent cases, respectively and decreased over time during the investigated period (Fig. 1). The results of the multivariable adjustment for predictors of the shift from VKA to NOACs are presented in Table 3. Independent predictors included female gender, southern district, higher CHADS_2_, and CHA_2_DS_2_-VASC scores, younger age, non-smoking, and treatment with antiplatelets upon diagnosis. Figure 1 presents the rates of shifting from NOACs to VKAs among the two study groups. These rates have decreased over time from 8% to 4.5% in 2012 to 0.5% and 0.7% in 2015 in the incident and prevalent groups, p< 0.001, respectively (Fig. 2).

**Table 2:** Comparison of baseline and clinical characteristics between patients who shifted versus those who did not switch to NOACs during follow-up

	Shift to NOACs during follow-up		
	Shifted N = 3737 % (n)	Did not Shift N = 3,877 % (n)	*p*-value
Age (years), Mean (SD)	73.5 (8.8)	73.3 (11.2)	**< 0.001**
Median (IQR)	69.3 (62.6; 75.6)	68.3 (60.2; 75.7)	
Female sex	47.9% (1791)	42.9% (1662)	**< 0.001**
Immigration ≥ 1990	39.6% (1481)	38.5% (1494)	0.339
SES score, Mean (SD)	5.85 (1.76)	5.90 (1.79)	0.207
Median (IQR)	6 (5; 7)	6 (5; 7)	
District			
North	17.7% (661)	18.9% (734)	**< 0.001**
Sharon	17.1% (639)	17.7% (685)	
South	22.1% (827)	18.3% (708)	
Center	18.7% (700)	21.0% (813)	
Jerusalem/ Hashfela	24.4% (910)	24.2% (937)	
CHADS_2_ score Mean (SD),	2.29 (1.21)	2.18 (1.36)	**< 0.001**
Median (IQR)	2 (1; 3)	2 (1; 3)	
CHADS_2_ risk levels			
0	3.9% (146)	9.1% (352)	**< 0.001**
1	22.2% (831)	24.1% (934)	
≥ 2	73.9% (2760)	66.8% (2591)	
CHA_2_DS_2_ VASC score Mean (SD)	3.92 (1.59)	3.68 (1.80)	**< 0.001**
Median (IQR)	4 (3; 5)	4 (2; 5)	
Baseline comorbidity			
Congestive heart failure	18.0% (672)	23.3% (902)	**< 0.001**
Ischemic heart disease	34.1% (1275)	35.4% (1371)	0.265
Myocardial infarction	13.8% (516)	14.6% (566)	0.339
IHD non-MI	23.3% (870)	23.7% (919)	0.683
Cerebrovascular accident	13.8% (514)	13.3% (516)	0.593
Transient ischemic attack	7.4% (276)	6.7% (260)	0.265
Peripheral arterial disease	7.5% (279)	8.2% (317)	0.266
Chronic kidney disease	46.3% (1732)	43.6% (1690)	0.017
Diabetes mellitus	40.4% (1509)	33.4% (1293)	**< 0.001**
Hyperlipidemia	88.8% (3318)	83.2% (3224)	**< 0.001**
Hypertension	87.9% (3284)	79.9% (3097)	**< 0.001**
Chronic obstructive pulmonary disease	2.9% (110)	3.2% (123)	0.608
Medications			
ACE inhibitors	35.4% (1322)	33.2% (1287)	0.1
ARBs	52.2% (1950)	48.3% (1871)	**0.048**
Beta blockers	20.3% (760)	17.7% (685)	**0.001**
Ca blockers	45.6% (1703)	44.0% (1705)	**0.003**
Diuretic	13.8% (515)	11.7% (455)	0.169
Nitrates	1.0% ( 36)	1.0% ( 37)	**0.008**
Other antiplatelets	4.1% (152)	2.6% ( 99)	0.999
Aspirin	30.3% (1133)	25.6% (993)	**< 0.001**
Incident 2012–2015	34.1% (1274)	27.9% (1081)	**< 0.001**

For prevalent cases, the status of all time-dependent factors is shown for 01/01/2012. For incident cases 2012–2015, the status at index date is shown

VKA: vitamin K antagonists, NOAC: new oral anticoagulants, SES: socioeconomic status, ACE: angiotensin converting enzyme, ARB: angiotensin receptor blocker, SD: standard deviation, CHADS: congestive heart failure, hypertension, age>75, diabetes mellitus, stroke/transient ischemic event; scale of 0 (lowest risk) to 6 (highest risk). CHA2DS2-VASc: Congestive heart failure, hypertension, age, diabetes mellitus, stroke/transient ischemic event, sex, vascular disease history; scale of 0 (lowest risk) to 9 (highest risk)

Bold means statistically significant, i.e. *p* < 0.05

**Table 3:** Mutually adjusted factors associated with shift to NOACS

	OR (95% CI)	p-value
Age per 1 year increment	0.992 (0.987; 0.997)	**0.003**
Female sex	1.18 (1.07; 1.29)	**0.001**
Smoking		
Never	1.0 (ref.)	
Ever	0.71 (0.61; 0.84)	**< 0.001**
Unknown	0.47 (0.31; 0.72)	**< 0.001**
District of inhabitance		
North	1.0 (ref.)	
Sharon	1.10 (0.94; 1.28)	0.240
South	1.30 (1.13; 1.51)	**< 0.001**
Center	1.01 (0.87; 1.17)	0.934
Jerusalem/ Hashfela	1.11 (0.97; 1.28)	0.138
*Baseline comorbidity*		
CHADS_2_ score		
**0**	**1.0 (ref.)**	
**1**	**2.15 (1.72; 2.69)**	**< 0.001**
**≥ 2**	**2.81 (2.23; 3.53)**	**< 0.001**
Hyperlipidemia	1.45 (1.26; 1.66)	**< 0.001**
Concomitant medications		
ARBs	1.26 (1.14; 1.39)	**< 0.001**
Antithrombotics	1.15 (1.03; 1.29)	**0.014**
Ca channel blockers	0.92 (0.83; 1.01)	0.090
Diuretics	0.84 (0.72; 0.98)	0.024
Nitrates	0.63 (0.39; 1.02)	0.062
Incident 2012–2015	1.73 (1.52; 1.98)	**< 0.001**

*NOAC* new oral anticoagulants, *SES* socioeconomic status, *ACE* angiotensin converting enzyme, *ARB* angiotensin receptor blocker, *SD* standard deviation, *CHADS* congestive heart failure, hypertension, age>75, diabetes mellitus, stroke/transient ischemic event; scale of 0 (lowest risk) to 6 (highest risk). *CHA2DS2-VASc* Congestive heart failure, hypertension, age, diabetes mellitus, stroke/transient ischemic event, sex, vascular disease history; scale of 0 (lowest risk) to 9 (highest risk)

Bold means statistically significant, i.e. *p* < 0.05

## Discussion

The current study evaluated the initiation and shifting patterns and timelines of NOACs vs. VKAs from a large healthcare provider in Israel. The main findings include the following: (1) A consistent increase in the rate of NOAC prescription and decrease in VKA prescription between 2012 and 2015. (2) The period from diagnosis to first dispensing was longer in NOACs than VKAs and decreased among patients treated with NOACs during the study period. (3) Shifting from a VKA to a NOAC was common (49%), occurring after a shorter time among the incident than prevalent cases, respectively, and decreased (the interval) throughout the study period. (4) Main predictors of such a shift were female gender, younger age, CHADS_2_ and CHA_2_DS_2_-VASC score, non-smoking, and antiplatelet therapy. (5) Shifting from a NOAC to a VKA occurred more frequently in incident cases and decreased over time.

The results of this study were stratified by two subgroups of AF (incident and prevalent cases). The baseline characteristics of these populations were different with incident cases, being younger and with lower risk for stroke as reflected by their CHA_2_DS_2_-VASc score. This finding can be explained by the increasing interest and awareness for stroke prevention seen with time and the emergence of NOACs as alternatives for VKA. The observed rates of initiation of NOACs throughout the entire study, as well as the significant increase in the proportion of eligible patients prescribed with NOACs, are consistent with previous literature (for an equivalent period) and are in line with the current guideline recommendation [1, 2, 16, 20–23, 27, 31–37]. However, consistent with previous studies, a significant proportion of patients are still not being prescribed an OAC for stroke prevention [27, 38–40].

The time from diagnosis to initiation of VKA therapy found in the current study was shorter than for NOACs and similar to the period reported by Khurshid et al. [9], where 86% of patients received a VKA. It is noteworthy that the stroke rate is substantial in the period between AF diagnosis and the administration of an oral anticoagulant (OAC) [9]. The delayed dispensing of NOACs in our study could perhaps stem from a more intricate approval process by the insurer, which has improved over the years, probably explaining the shortening of this gap. Other reported factors associated with delayed OAC dispensing include female gender, absence of hypertension, previous fall, and chronic kidney disease [9].

We found a significant shifting rate of about 50% from a VKA to a NOAC, more prominent in incident vs. prevalent cases. This rate is significantly higher compared to a study by Fosbol et al. [26] during a similar period (30%) and from those reported by Hale et al. [19] during 2010-2015. This discrepancy could result from differences in healthcare systems, particularly insurance and funding issues, such as the NOAC extended funding in our country. However, it is worth mentioning that although treatment guidelines recommend NOAC initiation over VKA in AF, routine shifts in stable VKA-experienced patients are not recommended [16]. The independent predictors of shifting from VKAs to NOACs are overall consistent with a previous report [26], mainly those comprising a higher CHADS_2_ and CHA_2_DS_2_-VASC score (except younger age) and an incident case rather than a prevalent case. These factors probably stem from a greater benefit of shifting with increased risk of stroke and from technical issues associated with insurance coverage (NOACs covered for higher CHADS_2_ score). Furthermore, when exploring etiologies for shifting from a VKA to NOAC, Hellfritzsch, et al., through a large cohort study, demonstrated that, at least in one out of five patients, shifting from VKA to NOAC therapy was preceded by a clinical event and subsequent need for medication re-adjustment [41]. Hale and colleagues reported that the main reason for shifting from VKA to NOAC treatment was NOACs being easier to use and manage [19]. The present study demonstrated that for patients on VKA shifting to NOAC, the time elapsed from initiation to shift was significantly longer in prevalent cases than newly-diagnosed patients and decreased in the latter group. This time lag may reflect the reluctance of physicians to switch an experienced and stable patient to NOACs.The shortening period in the incident (newly diagnosed) cases probably reflects the recommendation of NOACs as the drugs of choice in most patients with AF and the increased insurance coverage for this treatment in our country. Interestingly, a relatively novel finding was a non-negligible shifting from a NOAC to a VKA, which occurred more frequently in incident cases and decreased over time. The reasons are unknown but could result from contraindications developments for NOACs (mechanical valve implantation), adverse events, and financial limitations. The latter two, which can be more easily resolved with increased funding and types of NOACs, could explain the observed decrease trend in this shift over time.

The present study has several limitations. First, it is retrospective and observational, hence subject to inherent limitations of such a design. Specifically, patients’ preferences, which play a significant role in a real-world setting, could not have been evaluated. Second, diagnoses were primarily obtained from administrative databases (based on ICD-9), accordingly could be biased by coding errors. However, MHS continuously implements quality assurance measures. Third, the period for patient enrollment (2012–2015) might be considered limited for extensive, contemporary trends evaluation. Fourth, the entire exclusion of patients with a valvular disease might have excluded patients eligible for OACs. Fifth, OACs may have been prescribed to patients for indications other than AF (i.e., pulmonary embolism), and we lack the data to account for that. Yet, in the incident AF group, we included patients that were naïve to anticoagulation; in the prevalent AF group, we excluded patients with pulmonary embolism within six months from the AF. Thus, we believe that the potential bias is minimal. The fact that the included patients were diagnosed with AF and were eligible for OAC by their risk profile suggests that OAC prescription was aimed for stroke prevention in this population. Sixth, the different subtypes of NOACs and respective trends were not evaluated separately. Lastly, the Israel Ministry of Health co-sponsors medications purchase and co-payment of NOAC had changed over the years and was not identical to all patients due to different health plans, which could introduce bias. However, by including SES in our analyses, we believe it accounts for a partial adjustment for these differences.

## Conclusions

A consistent trend of increase in the rate of NOAC dispensing and decrease in VKA dispensing was observed over time. The period from diagnosis to first anticoagulant dispensing was longer in NOACs compared with VKAs and decreased in both throughout the investigated period. Shifting from VKAs to NOACs occurred in 50% of the cases, more frequently among incident cases, with a decreasing time from therapy initiation to switching. The main predictors of such a shift were female gender, younger age CHADS_2_, and CHA_2_DS_2_-VASC score, non-smoking and concomitant antiplatelet therapy. Shifting from a NOAC to a VKA occurred to a lesser extent, more frequently in incident cases, and decreased over time.

## Clinical implications

Upon diagnosis of AF or evaluation of a new patient with AF, prompt consideration of eligibility and indication and the patient-specific risk of OAC in general and NOAC specifically (as the class of choice in most patients) should be performed. When a decision to initiate therapy is made, it should be implemented with minimum delay. Caregivers and decision-makers should be aware of the reported shifting patterns when considering the initiation of NOACs. A socially and economically sensitive program to optimize the initiation and shifting of OAC therapy could significantly impact patient care and outcomes.

## Supplementary Information

**Additional file 1:** Supplementary appendix.

## Data Availability

The data that support the findings of this study are available from Maccabi Health Services, but restrictions apply to the availability of these data, which were used under license for the current study, and so are not publicly available. Data are however available from the authors upon reasonable request and with permission of Maccabi Health Services.

## Abbreviations

AF: Atrial fibrillation
ICD-9: International Classification of Diseases version 9
MHS: Maccabi Healthcare Services
NOACs: Non-Vitamin K antagonist oral anticoagulants
OAC: Oral anticoagulant
VKAs: Vitamin K antagonists
