# Supplementary material for: Shifting from vitamin K antagonists to non-vitamin K antagonist oral anticoagulants in patients with atrial fibrillation: predictors, patterns and temporal trends
Source: BMC Cardiovasc Disord. 2021 Oct 13;21:493. doi: 10.1186/s12872-021-02295-w (PMC8513259; doi:10.1186/s12872-021-02295-w)
Supplement: Supplementary file 1 — Additional file 1: Supplementary appendix. [file 12872_2021_2295_MOESM1_ESM.docx]

**Figure 1.** Temporal trends in first medication prescribed/dispensed among incident NVAF


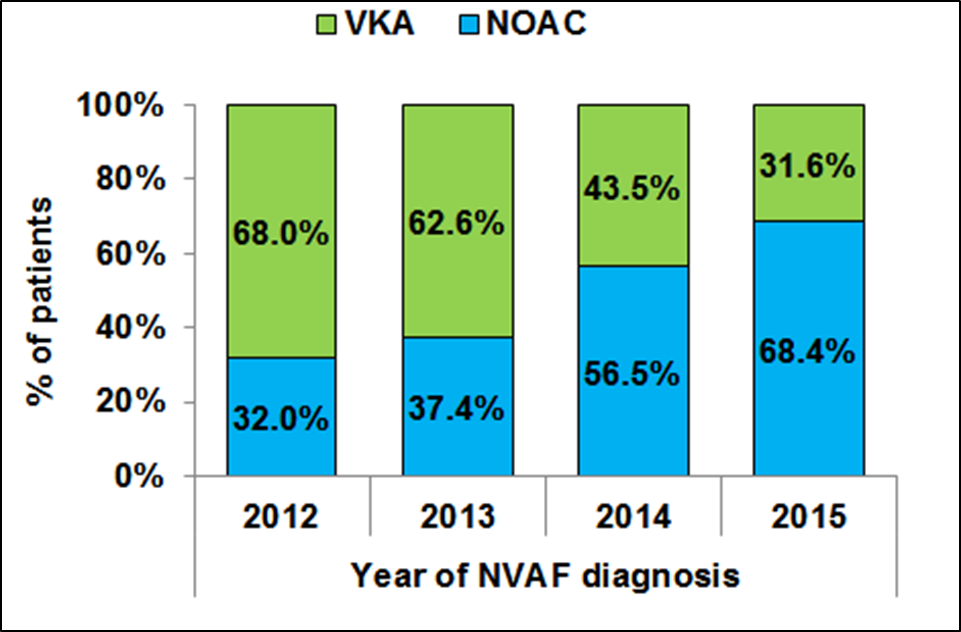


P<0.001

VKA- vitamin K antagonists; NOAC- New antagonist oral anticoagulants

**Figure 2.** Time (months, median [IQR]) from diagnosis to first dispense of therapy among incident patients according to the year of diagnosis
